# Evaluation and characterization of HSPA5 (GRP78) expression profiles in normal individuals and cancer patients with COVID-19

**DOI:** 10.7150/ijbs.54055

**Published:** 2021-02-18

**Authors:** Jiewen Fu, Chunli Wei, Jiayue He, Lianmei Zhang, Ju Zhou, Kyathegowdanadoddi Srinivasa Balaji, Shiyi Shen, Jiangzhou Peng, Amrish Sharma, Junjiang Fu

**Affiliations:** 1 Key Laboratory of Epigenetics and Oncology, the Research Center for Preclinical Medicine, Southwest Medical University, Luzhou 646000, Sichuan, China.; 2 Department of Pathology, the Affiliated Huaian No. 1 People's Hospital of Nanjing Medical University, Huai'an 223300, Jiangsu, China.; 3 PG Department of Biotechnology, Teresian College, University of Mysore, Mysore 570011, Karnataka, India.; 4 Department of Thoracic Surgery, The Third Affiliated Hospital of Southern Medical University, Guangzhou 510000, China.; 5 Department of Experimental Radiation Oncology, The University of Texas MD Anderson Cancer Center, Houston 77030, Texas, USA.

**Keywords:** cancer, HSPA5, prognostics, SARS-CoV-2, transcriptomics.

## Abstract

HSPA5 (BiP, GRP78) has been reported as a potential host-cell receptor for SARS-Cov-2, but its expression profiles on different tissues including tumors, its susceptibility to SARS-Cov-2 virus and severity of its adverse effects on malignant patients are unclear. In the current study, HSPA5 has been found to be expressed ubiquitously in normal tissues and significantly increased in 14 of 31 types of cancer tissues. In lung cancer, mRNA levels of* HSPA5* were 253-fold increase than that of *ACE2*. Meanwhile, in both malignant tumors and matched normal samples across almost all cancer types, mRNA levels of *HSPA5* were much higher than those of *ACE2*. Higher expression of *HSPA5* significantly decreased patient overall survival (OS) in 7 types of cancers. Moreover, systematic analyses found that 7.15% of 5,068 COVID-19 cases have malignant cancer coincidental situations, and the rate of severe events of COVID-19 patients with cancers present a higher trend than that for all COVID-19 patients, showing a significant difference (33.33% vs 16.09%, *p*<0.01). Collectively, these data imply that the tissues with high HSPA5 expression, not low ACE2 expression, are susceptible to be invaded by SARS-CoV-2. Taken together, this study not only indicates the clinical significance of HSPA5 in COVID-19 disease and cancers, but also provides potential clues for further medical treatments and managements of COVID-19 patients.

## 1. Introduction

Heat shock protein family A (Hsp70) member 5 (HSPA5) (OMIM: 138120), also called binding immunoglobulin protein (BiP) or glucose regulating protein 78 (GRP78), is a protein, that in humans, is encoded by the *HSPA5* gene. HSPA5 is commonly positioned in the endoplasmic reticulum (ER). When the ER is stressed, HSPA5 can translocate to the nucleus, the mitochondria and cell surface complexing with other proteins. On the cell surface, HSPA5 plays a multi-functional role in cell proliferation, cell viability, apoptosis, and regulation of innate and adaptive immunity 1. HSPA5 is the master chaperone protein for unfolded protein response for ER function when unfolded or misfolded proteins accumulated 2. HSPA5 involves in the correct folding and degradation of misfolded proteins through interacting with DNAJC10/ERdj5, facilitating DNAJC10/ERdj5 release from substrates. Dysregulations of these stress proteins including HSPA5 are associated with many human diseases including cancers, immunological diseases, cardiovascular diseases, neurodegenerative diseases, obesity, stroke and infectious diseases 3-6. Targeting HSPA5 may be potential in therapy for human diseases 7, 8 as well as COVID-19 (Coronavirus Disease 2019) 9-11.

SARS-Cov-2 (severe acute respiratory syndrome coronavirus 2), which causes the disease of COVID-19, is a member of beta coronaviruses like the previous coronaviruses SARS (severe acute respiratory syndrome) and MERS (middle east respiratory syndrome). COVID-19 virus causes global pandemic events since the first outbreak in Wuhan of Hubei, China in late December 2019. As of the January 11, 2021, the total confirmed cases are 90,833,894, and deaths are 1,942,974 worldwide based on the report from the Center for Systems Science and Engineering (CSSE) at Johns Hopkins University (https://coronavirus.jhu.edu/). The host cell entry of coronavirus was regulated by the viral spike protein ( ~1300 amino acids), originate in homotrimeric state over the virion particle and characterize coronaviruses 12, 13. Various host cell receptors or entry related-proteins are identified for different coronaviruses such as heparan sulfate proteoglycans, angiotensin-converting enzyme 2 (ACE2), transmembrane protease serine 2 (TMPRSS2), aminopeptidase N, HSPA5, furin, and O-acetylated sialic acid 14-17. Molecular chaperones are involved in multiple pathophysiological processes including viral infection by spike protein attacks 18. The cell-surface receptor HSPA5 is susceptible to viral recognition through the substrate-binding domain (SBD), thereby mediating the virus entry into the cells 10, 19, 20. The spike binding site to HSPA5 is predicted by molecular model docking and structural bioinformatics, and revealed that the binding is more favorable at the regions III (C391-C525) and IV (C480-C488) in the spike protein 15, and region IV is the major driving force for HSPA5 binding which may be useful for developing therapeutics specific against COVID-19. Indeed, recently virtual screening studies revealed that known HSPA5 inhibitors interfere with SARS-Cov-2 infection 21. Thus HSPA5 may be a receptor for SARS-CoV-2 attachment and entry 10, 15, 22, 23. The expression levels of HSPA5 were found to be higher in the SARS-COV-2-positive group compared to the other groups 24. Pep42, a cyclic peptide, binds to HSPA5 at the surface of cancer cells 25, 26.

The expression levels of HSPA5 in different tissues might closely related to the susceptibility and severity of the viral infection. Organ dysfunctions, such as shock, acute cardiac injury, acute respiratory distress syndrome (ARDS), acute kidney injury (AKI), and death can occur in severe events of COVID-19 disease 27, 28. Older people with comorbidities, such as high blood pressure, diabetes, cardiovascular disease and cerebrovascular disease have been reported to affect the COVID-19 severity 29, 30.

Patients with malignant cancers affected survival status and gene expression in tumor tissues. The incidence of malignant cancers is getting higher and higher, and recently was found to be the common comorbidity of COVID-19. Although one study showed that there was no significant difference in the severity of COVID-19 in cancer patients, as a receptor of SARS-Cov-2 31, dysregulation of HSPA5 expression in cancer patients' tissues, particularly in the lungs, should affect the susceptibility and severity of this virus infection. Targeting HSPA5 may help to develop and design novel therapeutic strategies against virus infections 32 including SARS-Cov-2, which might also associate with human carcinoma between endoplasmic reticulum stress and anti-viral activities 23, 33. Hence understanding of the HSPA5 expression profiles on different normal tissues and malignant tumors is important. But the tumor patients with its receptor expression files for HSPA5 in this outbreak have not been reported. In this study, the differences in HSPA5 expression in various types of normal and cancer tissues were evaluated. The influences of these differences on the impacts of SARS-CoV-2 infections were dissected. The cancer patients with COVID-19 were also estimated to assess the susceptibility and severity.

## 2. Materials and methods

### 2.1. Sources for data analysis and ethical concerns

The mRNA and protein expressions for HSPA5 from different normal tissues were obtained in the database of the Human Protein Atlas (HPA) (https://www.proteinatlas.org/ENSG00000044574-HSPA5) 34, 35. The immunohistochemistry (IHC) or immunofluorescence (IF) images of HSPA5 (Ensembl ID: ENSG00000044574.7) were also gained from the HPA database (https://www.proteinatlas.org/ENSG00000044574-HSPA5/cell), (https://www.proteinatlas.org/ENSG00000044574-HSPA5/pathology), or (https://www.proteinatlas.org/ENSG00000044574-HSPA5/cell#human), respectively 36, 37. The expressions of HSPA5 were verified using Genotype Tissue Expression (GTEx) projects. The Gene Expression Profiling Interactive Analysis (GEPIA) dataset (http://gepia.cancer-pku.cn) or GEPIA 2 (http://gepia2.cancer-pku.cn/#index), an updated and enhanced version of GEPIA, which were developed recently 38, and ONCOMINE (https://www.oncomine.org), were used to compare the expressions between tumors and normal tissues. FANTOM5 database come from https://fantom.gsc.riken.jp/5/. The NCBI database (https://www.nih.gov/) was used. All datasets and clinical data for COVID-19 patients infected with SARS-CoV-2 were retrieved from the published literatures with statements of written informed consent. Thus no local ethics committee was required to approve this study.

### 2.2. Homology analysis

Homologs for HSPA5 were conducted by the NCBI program (https://www.ncbi.nlm.nih.gov/homologene/?term=Homo+sapiens+HSPA5) 39.

### 2.3. HPA analysis for HSPA5

The *HSPA5* expressions in mRNA and protein were analyzed differentially in human normal and tumor tissues from the HPA database, which includes IHC-based expression for approximately 20 different types of common cancers in 216 cancer patients (maximum 12 patients in a group) 37. The mRNA levels for *HSPA5* in different normal tissues were obtained from the consensus datasets of three sources (HPA, GTEx and FANTOM5). Consensus normalized expression levels for 54 tissue types and 7 blood cell types were created from the above three datasets with the normalization pipeline (https://www.proteinatlas.org/about/assays+annotation#normalization_rna). Protein expression data were shown for each of the 44 normal tissues. Two antibodies for HSPA5 (cat #: CAB005221, sc-1050, Santa Cruz Biotechnology; or cat #: HPA038845, Sigma-Aldrich) were used for IHC staining in these data 40.

### 2.4. GEPIA analysis for HSPA5 and verification

The mRNA expressions of* HSPA5* in tumors and normal tissues were analyzed in the GEPIA dataset, for analyzing the RNA sequencing (RNA-seq) expression data of 9,736 tumors and 8,587 normal samples from the Cancer Genome Atlas and GTEx projects, using a standard processing pipeline 38. The gene expressions of HSPA5 in cancers and those in normal samples were verified by using ONCOMINE databases. HSPA5 expressions for samples in overall survival (OS) analysis were divided into high and low of two groups using a median expression, and analyzed by a Kaplan-Meier survival plot using the log-rank test. Logrank *p* < 0.01 was considered as significant differences.

### 2.5. Systematic reviews of malignant tumors in COVID-19 patients infected with SARS-CoV-2

We searched PubMed, Medline, and Google Scholar on November 25, 2020 from published studies describing the clinical characteristics of COVID-19 due to SARS-CoV-2 and cancers. The search terms “cancer” and “2019-nCoV” or “COVID-19” with no time restrictions were performed. The related works of literature were screened and analyzed, clinical signs and symptoms caused by COVID-19 and cancers were collected, and studies describing patients' malignant cancer status were conducted. The number of patients and the rate of severity combined with malignant cancers were calculated. Studies of incomplete symptom descriptions were excluded. The patient severe events were defined as the admission to ICU, requiring mechanical ventilation, or death of COVID-19. All the selected articles were analyzed by two independent investigators.

## 3. Results

### 3.1. HSPA5 is highly conserved

Homologs of the HSPA5 protein showed that it is highly conserved in different species, including H.sapiens, chimpanzee, Rhesus monkey, mouse, dog, cow, rat, chicken, zebrafish, fruit fly, mosquito, C.elegans, S.cerevisiae, K.lactis, E.gossypii, S.pombe, M.oryzae, N.crassa, A.thaliana, rice, and frog (Supplementary figure 1A&B). This implied that HSPA5, similar to ACE2 in animals of different species 39, 41, have the potentials to bind to the receptor binding domain (RBD) of the spike glycoprotein, making it a probable natural host of SARS-CoV-2.

### 3.2. Expression of HSPA5 in normal tissues

**Figure 1:**
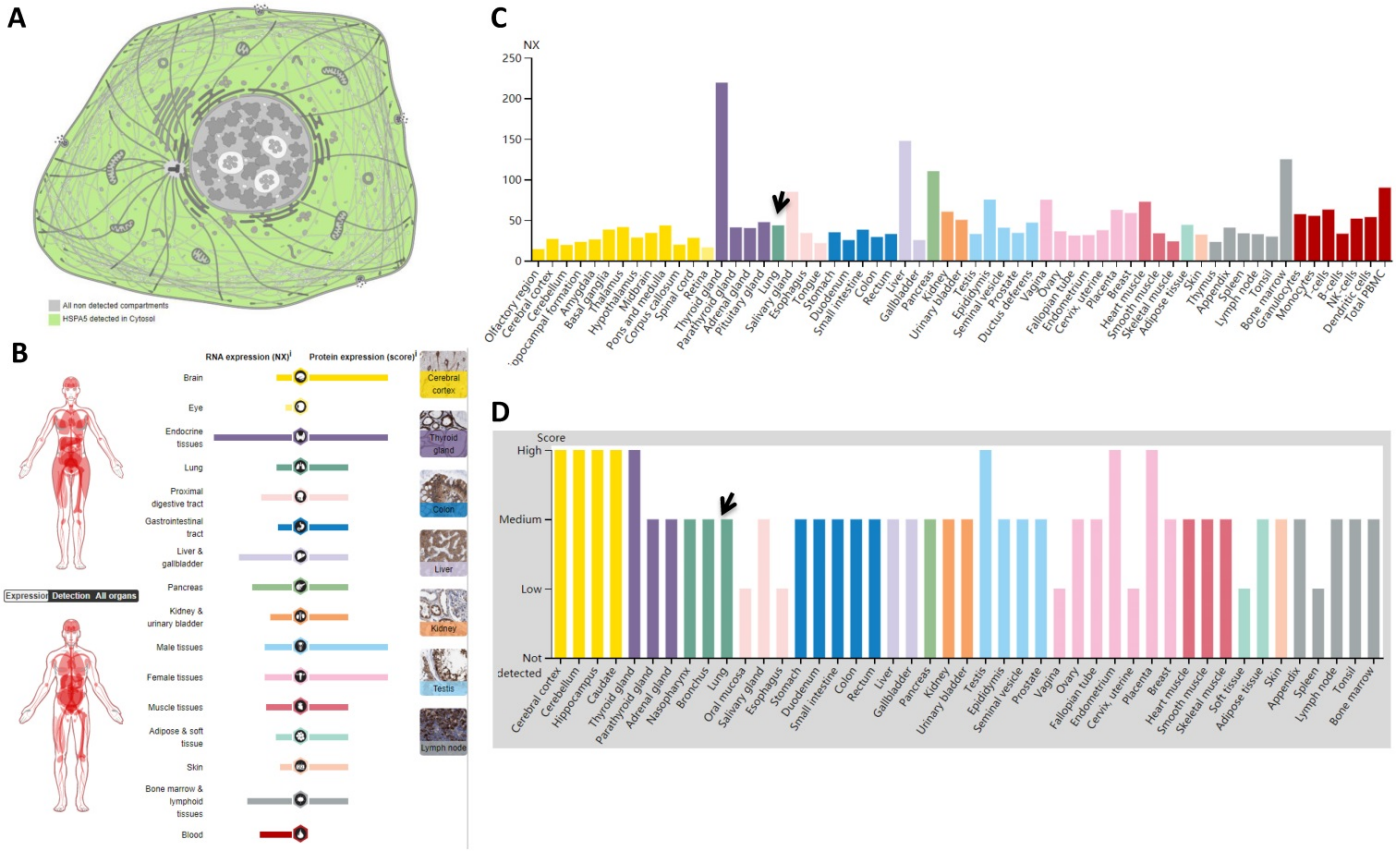
Localizations and expressions of HSPA5 in normal tissues. A. Cellular localization of the HSPA5 protein. Green in color indicates HSPA5 detected in cytoplasm, whereas gray in color indicates the absence. B. The summary of mRNA and protein expressions of *HSPA5*. Color-coding columns are based on tissue groups, each consisting of tissues with functional features in common. The respective images for normal tissues with staining of HSPA5 protein in the HPA (scale bar 200 µm). C. The mRNA expressions of *HSPA5* in normal tissues. Consensus dataset of mRNA level are derived from HPA dataset, GTEx dataset, and FANTOM5 dataset. A NX value of 1.0 is defined as a threshold for *HSPA5* mRNA expression. D. The HSPA5 protein expressions in normal tissues from the HPA. Protein expression data are shown for each of the 44 tissues. Arrows indicate the lung tissue. HPA, Human Protein Atlas.

Subcellular locations from HPA data revealed that HSPA5 is localized to the cytoplasm (Fig. 1A). Expression of *HSPA5* in RNA level showed low tissue specificity, with highest in the thyroid gland (NX: 219.3) and lowest in the olfactory region (NX: 14.3) (Fig. 1B&C), and in protein level showed cytoplasmic expression ubiquitously, highly abundant in immune, neuronal cells and thyroid follicular cells, specifically from 8 tissues of the cerebral cortex, cerebellum, hippocampus, caudate, thyroid gland, testis, endometrium, and placenta (Fig. 1B&D). The other 31 tissues including the lungs showed medium levels of protein, and only 6 tissues showed low levels (Fig. 1D).

### 3.3. Expression of HSPA5 in normal lungs of humans

The main route of transmission of SARS-CoV-2 is by droplets from the respiratory tract, thus causing the severe acute respiratory syndrome. The expression levels of receptors in the lungs are important. However, from our previous study and other studies, ACE2 expression was very low in human lungs, showing moderate expression in the alveolar macrophages, and a few in the type I alveolar epithelial cells and type Ⅱ alveolar epithelial cells, but most of the typeⅡalveolar epithelial cells were ACE2 negative 39, 42. Thus we then investigated *HSPA5* mRNA expression on normal human lungs and found that, in the lung tissues, the mRNA level shows the NX value 43.6 (Fig. 1C, arrow) and the protein level is medium (Fig. 1D, arrow). This consensus 43.6 NX (Table 1) was derived from databases of LUNG- HPA RNA-seq, LUNG - GTEx RNA-seq (Supplementary figure 2A), and LUNG - FANTOM5 CAGE (Supplementary figure 2B).

The comparison of mRNA expressions of *ACE2* and *HSPA5* were conducted through analyzing datasets from HPA, GTEx, and FANTOM5 in human normal lungs, and found that mRNA levels of *HSPA5* is 54.4-fold higher than that of *ACE2* mRNA levels (Supplementary figure 2C), demonstrating that, in addition to ACE2, HSPA5 might play very important roles for SARS-CoV-2 entry.

**Figure 2:**
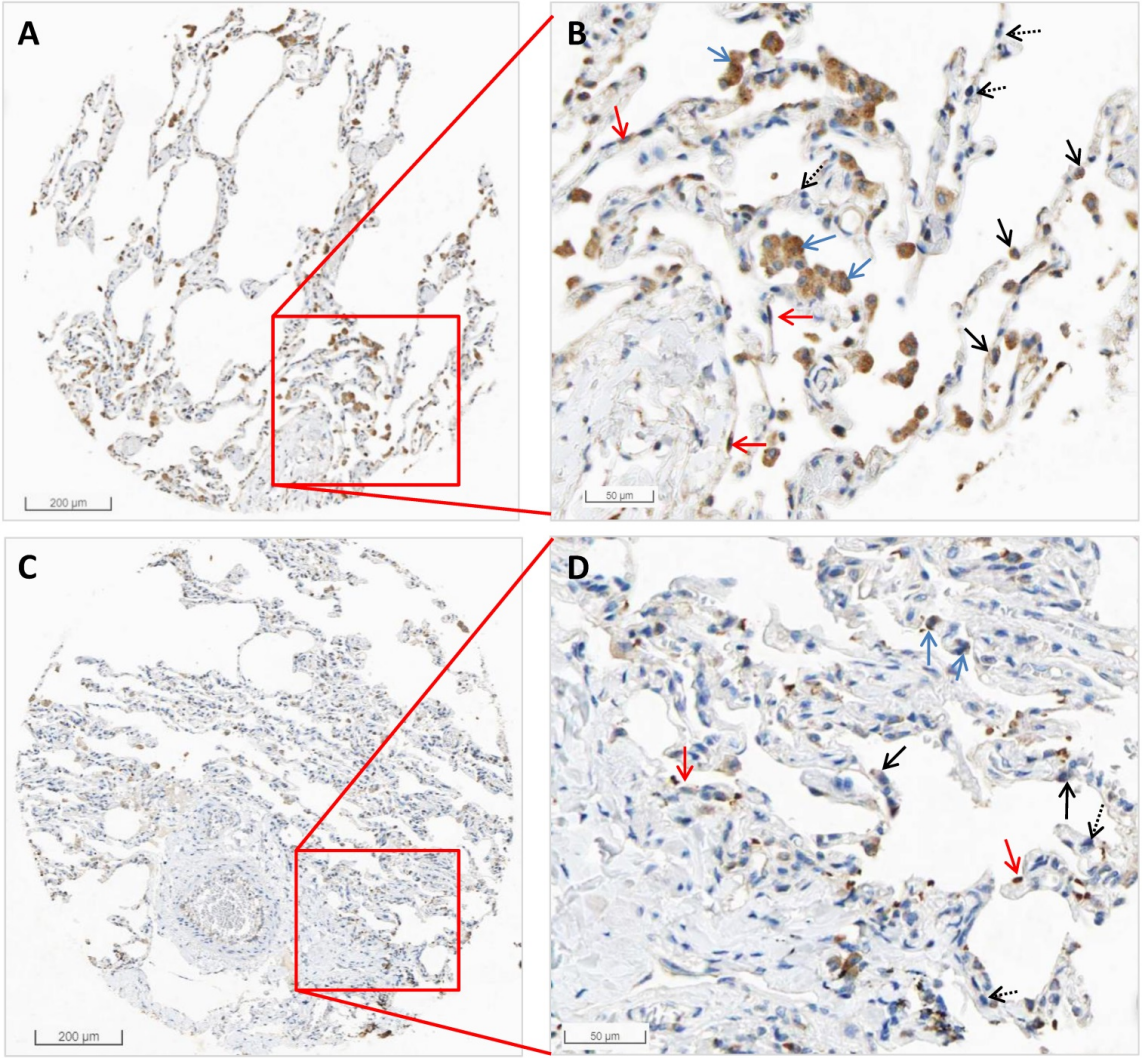
The representative images by IHC in normal tissues of the lungs of HSPA5. The protein expressions for HSPA5 from normal tissues were obtained in the database of the Human Protein Atlas (HPA) (https://www.proteinatlas.org/ENSG00000044574-HSPA5). A. IHC images from the tissue of normal lungs from a female of age 49 (Patient id: 2268). B. Enlarged picture from A. C. IHC images from the tissue of normal lungs from a male of age 21 (Patient id: 2101). D. Enlarged picture from C. Arrows in blue indicate the representative positive results for macrophages, arrows in red indicate the representative positive results for type I alveolar epithelial cells, arrows in black indicate the representative positive results for type Ⅱ alveolar epithelial cells (Fig.2C, black arrow), and arrows in dashed black indicate the representative negative staining for type Ⅱ alveolar epithelial cells. The scale bars for 200 µm and 50 µm are indicated.

From the HPA RNA-seq data, we found that the mRNA expression of *HSPA5* in pneumocytes accounts for 31.67%, endothelial cells 28.33%, macrophages 10%, bronchial epithelium 5%, and other cell types 25%. The results are shown in Table 2. Protein expression of HSPA5 by IHC in both macrophages and pneumocytes of lungs were medium. The representative IHC images of normal lung tissue are shown in Figure 2. From these results, we found that HSPA5 expressions using the HSPA5 antibody (cat# CAB005221) in alveolar macrophages are high and are mainly located in the cytoplasm and a few in the nucleus (Fig. 2B&D, blue arrows). Intensive positive staining of HSPA5 was localized at cytoplasm of type I alveolar epithelial cells (Fig. 2B&D, red arrows) and type Ⅱ alveolar epithelial cells (Fig. 2B&D, black arrow); but some of the typeⅡ alveolar epithelial cells were HSPA5 negative stained (Fig. 2B&D, dashed black arrows). Interestingly, different antibodies from HPA project showed slightly different results and found that the HSPA5 antibody (cat #: HPA038845) showed high expression in both macrophages and pneumocytes (Data not shown). Collectively, these IHC results for protein levels are consistent with those by RNA-seq for mRNA levels of *HSPA5*, showing much higher than that of *ACE2* 39, 43, 44.

### 3.4. Expression values of HSPA5 in malignant tumors and cancer cell lines

**Figure 3:**
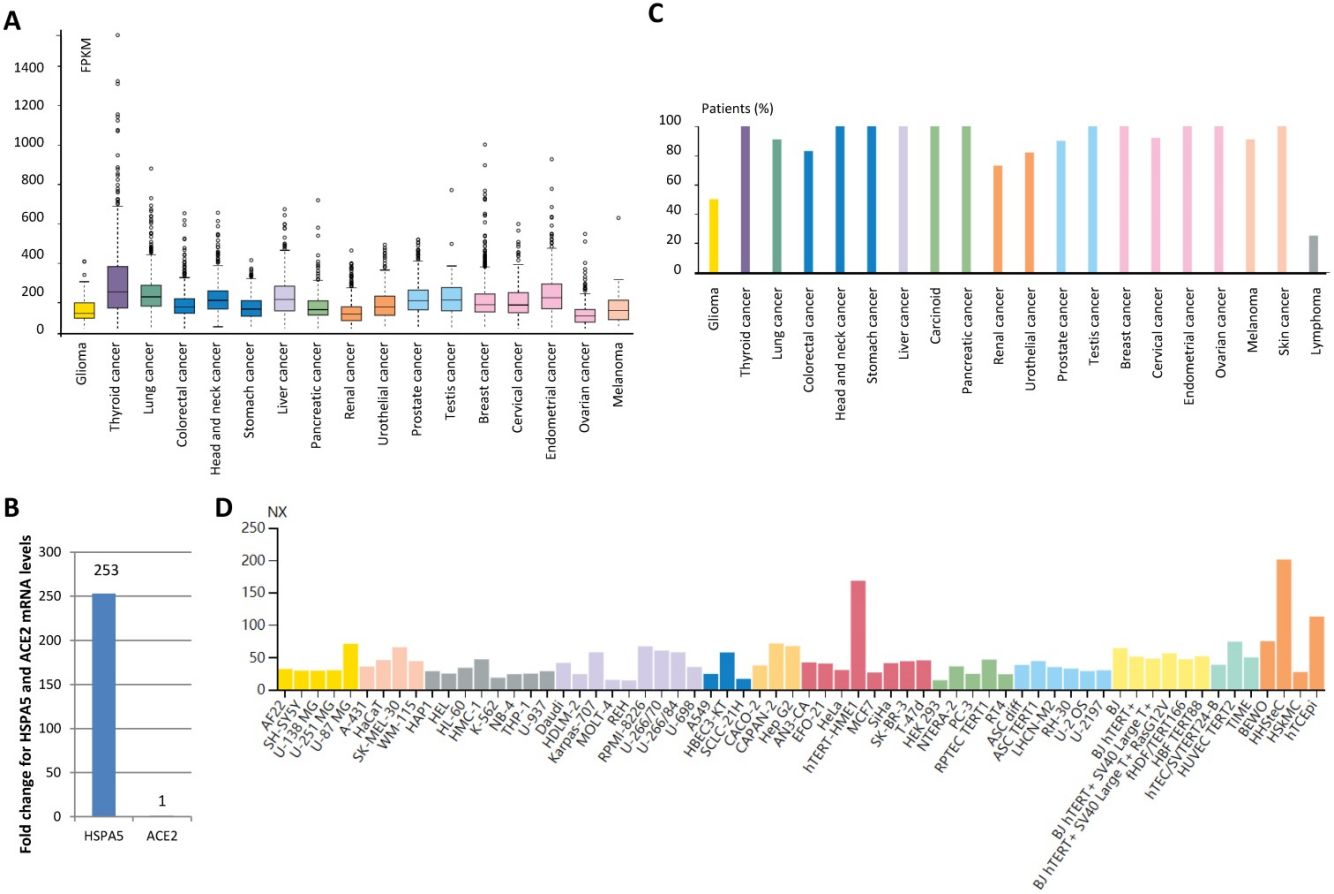
Expression value of HSPA5 in malignant tumor tissues and cancer cells. A. *HSPA5* RNA expression in malignant tumor tissues of different types of cancers. B. Comparison for *HSPA5* and *ACE2* mRNA levels in lung cancer by analysis of TCGA dataset. C. The protein expression of HSPA5. D. *HSPA5* RNA expression in the cancer cell lines. For each cancer type, color-coded bars indicate the percentage of patients (≤12 patients) with high and medium expressed of protein level. The cancer types are color-coded according to which type of normal organ the cancer originates from. The cell lines we analyzed are divided into 12 color-coded groups according to the organ from.

By analyzing patient malignant tissues of different types of cancers, we found that low tissue specificity for the *HSPA5* mRNA level is detected in all by RNA-seq (Fig. 3A). In order to test which genes changes more in lung cancer, we analyzed *HSPA5* mRNA and *ACE2* mRNA in TCGA dataset from 994 samples and found that *HSPA5* mRNA levels was 253-fold than that of *ACE2* (Fig. 3B), indicating that HSPA5 might play important roles for SARS-Cov-2 entry in cancer patients through lungs. This was supported partially by a systematic review of malignant cancers in COVID-19 patients that men and lung cancer patients were more likely to have COVID-19 when studied for ACE2 expression 31.

The protein expression of HSPA5 was found to be ubiquitously cytoplasmic high expressed, highly abundant in immune, neuronal cells, and thyroid follicular cells (Fig. 3C, Supplementary figure 3A~E). Malignant cells showed moderate to strong cytoplasmic staining (Supplementary figure 3F~I). Membranous staining was observed in a few cases of ovarian and pancreatic cancers (Data not shown). In the cancer cell lines, *HSPA5* RNA expressions were enhanced, compared to the matched normal tissues (Fig. 3D, data not shown).

### 3.5. The expression of *HSPA5* is higher in malignant tumors than that in matched normal samples

**Figure 4:**
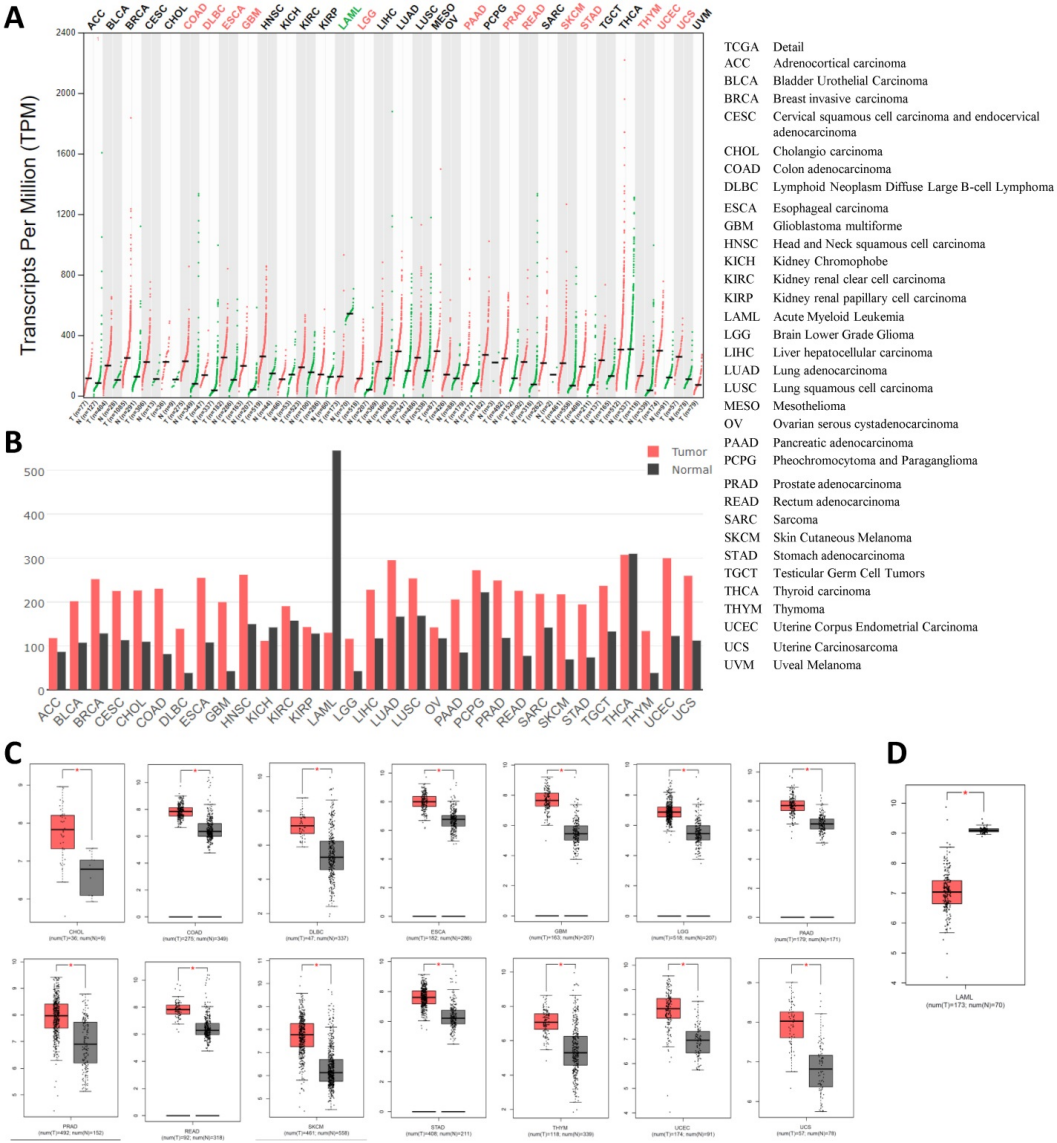
Expression values of *HSPA5* in malignant tumors and paired normal samples. A. The *HSPA5* expression profiles across all cancer samples and paired normal tissues by dot plots. B. The HSPA5 expression profiles across all cancer samples and paired normal tissues by bar plots. The height of bar represents the median expression of certain cancer type or matched normal tissue. C. *HSPA5* was overexpressed in fourteen cancer types by box plots. D. *HSPA5* was decreased in one cancer of LAML by box plots. *HSPA5* mRNA expressions in caners and matched normal tissues were gained from the dataset of GEPIA. GEPIA, Gene Expression Profiling Interactive Analysis. *, *p*<0.01.

Then, we compared to the *HSPA5* mRNA expression profile across all tumor samples and their paired normal tissues in 31 types of cancers using the GEPIA dataset. The results showed that all cancer tissues can express *HSPA5*, and the highest expression levels were noticed in thyroid carcinoma (Fig. 4A&B). The expressions of *HSPA5* were significantly increased in 14 types of cancers, including cholangio carcinoma (CHOL), colon adenocarcinoma (COAD), lymphoid neoplasm diffuse large B-cell lymphoma (DLBC), esophageal carcinoma (ESCA), glioblastoma multiforme (GBM), brain lower grade glioma (LGG), pancreatic adenocarcinoma (PAAD), prostate adenocarcinoma(PRAD), rectum adenocarcinoma (READ), skin cutaneous melanoma (SKCM), stomach adenocarcinoma (STAD), thymoma, uterine corpus endometrial carcinoma (UCEC), and uterine carcinosarcoma (UCS) (Fig. 4C, p<0.01). The expressions of *HSPA5* were significantly decreased only in acute myeloid leukemia (LAML) (Fig. 4D, p<0.01). But the levels of *HSPA5* in LAML of paired normal tissue are much higher than that of the highest one in the thyroid carcinoma tissue. The gene expressions of HSPA5 in tumors and those in normal samples were verified using the database of ONCOMINE (Data not shown). Other cancer types of tissues, except kidney chromophobe (KICH) and thyroid carcinoma (THCA), the expressions of *HSPA5* were increased but not significantly (Fig. 4, A&B). In addition, HSPA5 expressions in lung cancers were upregulated compared to normal tissues from TCGA dataset (Data not shown). Altogether, those results indicated that the HSPA5 might play more important roles for SARS-Cov-2 entry in most of the cancer patients through different malignant tissues, or be prone to attack in most of the different types of cancer patients.

### 3.6. The expressions of *HSPA5* are much higher than those of *ACE2* in both malignant tumors and matched normal samples

**Figure 5:**
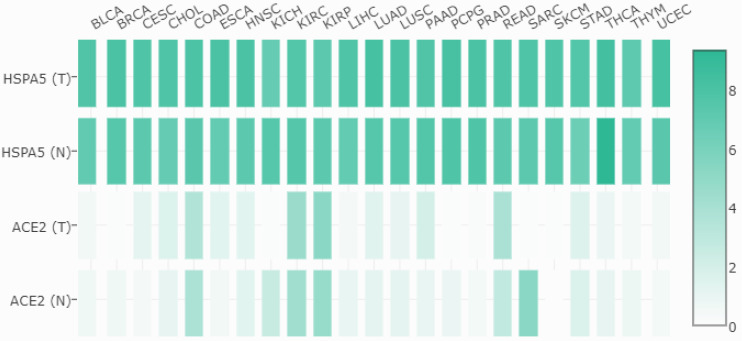
Expression comparisons between *HSPA5* and *ACE2* in both malignant tumors and matched normal samples in TCGA normal datasets. The cancer types are indicated on the top, and full names are shown in the figure 4. The gene for *HSPA5* with/without tumor are indicated as HSPA5 (T)/HSPA5(N), and for *ACE2* with/without tumor are indicated as ACE2 (T)/ACE2(N) on the left, respectively. The density of color in each block represents the median expression value of a gene in a given tissue, normalized by the maximum median expression value across all blocks. Different genes in same tumors or normal tissues can be compared in one plot, and the values can be obtained through online (http://gepia.cancer-pku.cn/detail.php?gene=HSPA5###).

Then, we compared the mRNA expressions between *HSPA5* and *ACE2* in both malignant tumors and matched normal tissues, and the results are shown in Figure 5. From these results, we found that, unlike *HSPA5*, the mRNA expressions of *ACE2* were significantly overexpressed in some types of cancers, including colon adenocarcinoma (COAD), kidney renal papillary cell carcinoma (KIRC), pancreatic adenocarcinoma (PAAD), rectum adenocarcinoma (READ) and stomach adenocarcinoma (STAD), but significantly lower expressions in other types of cancers including kidney chromophobe (KICH), sarcoma, testicular germ cell tumors (TGCT) and thyroid carcinoma (THCA), than those in normal tissues (Figure 5, and data not shown), which has been reported recently for *ACE2* 31. Surprisingly, the mRNA expressions of *HSPA5* are much higher than those of *ACE2* in both malignant tumors and normal samples across almost kinds of cancer types (Figure 5).

### 3.7. Prognostic values of *HSPA5* in malignant tumors

**Figure 6:**
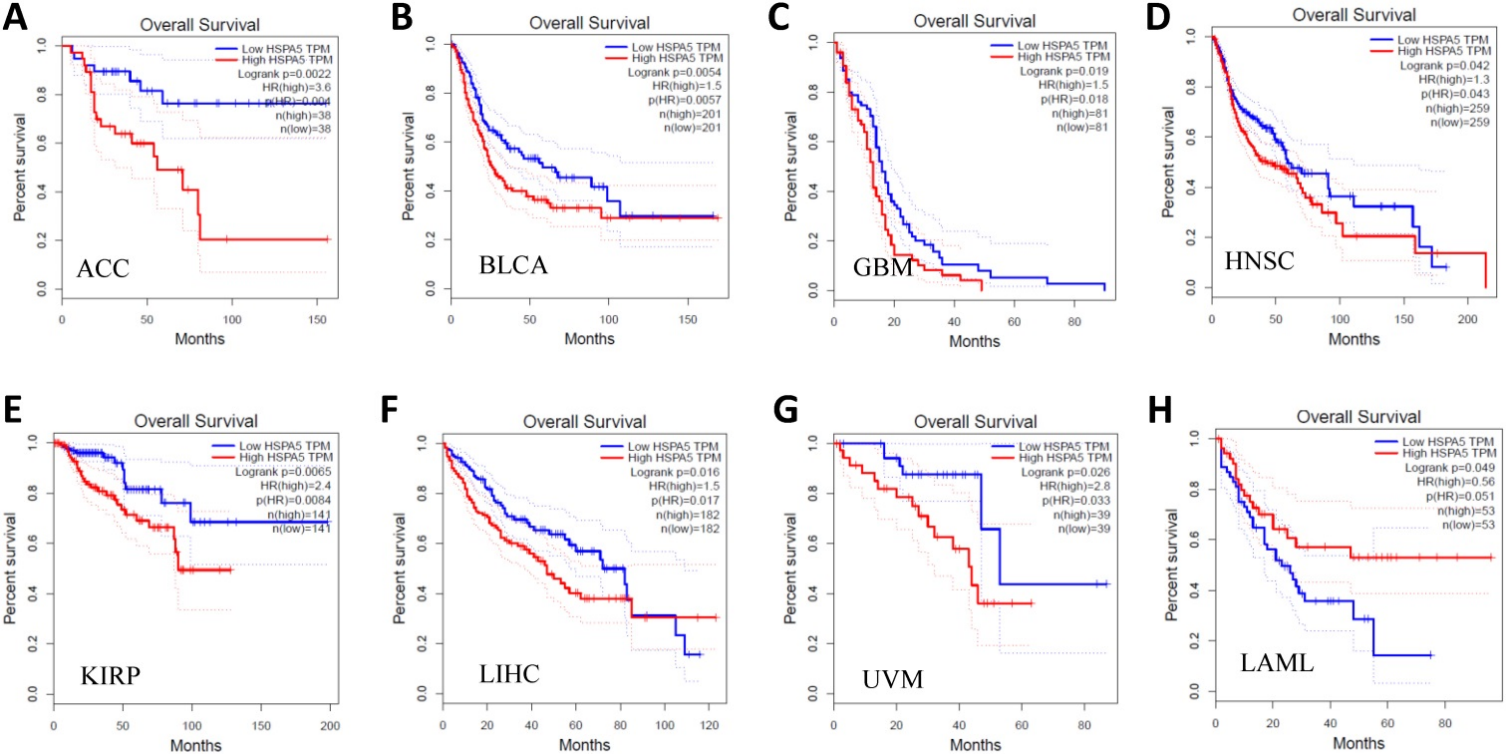
Prognostic values of *HSPA5* in pan-cancer. The prognostic value of *HSPA5* in eight cancer types from the GEPIA dataset. A~H. ACC, BLCA, GBM, HNSC, KIRP, LAML, LIHC, UVM, respectively. *, *P* < 0.01. The cancer types of full names are shown in Figure 4. GEPIA, Gene Expression Profiling Interactive Analysis. HR, Hazards Ratio.

After that, we further investigated the prognostic values of HSPA5 in a pan-cancer. Cancer patients in survival analysis were divided into high expressed and low expressed groups using median *HSPA5* expression and analyzed by overall survival (OS) Kaplan-Meier plots. The results are shown in Figure 6, and we found that the over-expressions of *HSPA5* significantly decreased patient OS in the indicated seven types of cancers, including adrenocortical carcinoma (ACC), breast invasive carcinoma (BLCA), glioblastoma multiforme (GBM), head and neck squamous cell carcinoma (HNSC), kidney renal papillary cell carcinoma (KIRP), liver hepatocellular carcinoma (LIHC), and uveal melanoma (UVM) (Fig. 6, A~G); whereas low expressions of *HSPA5* significantly decreased patient OS only in acute myeloid leukemia (LAML) (Fig. 6, H). These results indicate that overall survivals are reduced significantly in most types of malignant tumor patients when HSPA5 is overexpressed.

### 3.8. Characteristics of malignant cancer patients infected with SARS-CoV-2

**Figure 7:**
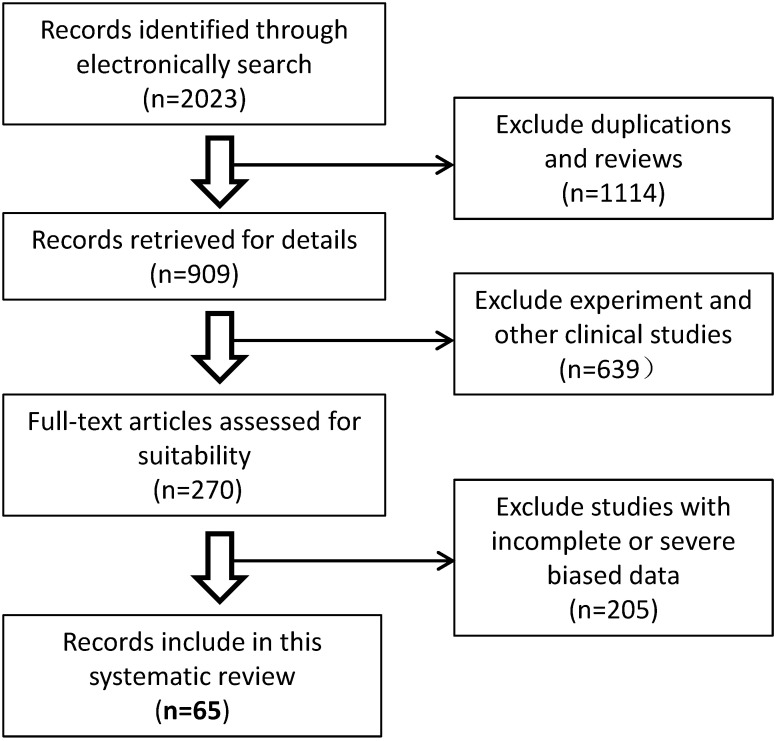
Schematic flow diagram for the selection of the included studies in the systematic review.

The recently published studies describing the clinical characteristics of COVID-19 and malignant cancers were screened and analyzed by systematic review. Overall, sixty-five published studies that evaluated patients' malignant cancer statuses were included from 2023 potentially relevant studies in our systematic review. A schematic flow diagram for the selection of the included studies with eligible trials and exclusion criteria is shown in Fig. 7. A total of 70,874 COVID-19 cases in China, USA, Belgium, Spain, Italy, España, Korea, Iran, Poland, Turkey, Germany, France, Turkey, UK, Switzerland, and Israel, or international multicenter were included. Among them, 5,068 COVID-19 cases (7.15%, 5068/708744) had comorbidities of malignant cancer (Table 3). Among these cancer patients, men and lung cancers were more likely to have COVID-19 (Data not shown). The rate of severe events for COVID-19 with malignant cancer patients was 33.33% (1689/5068), while the rate of severe events for all patients of COVID-19 was 16.09% (11404/70874) (Table 3), which is significantly higher for malignant patients with COVID-19 disease (33.33% vs 16.09%, *p*<0.01), suggesting overexpression of HSPA5 might contribute to the severity of COVID-19 patients. These data are consistent with higher expression of HSPA5 in tumor tissues from different types of cancer patients, but likely through different mechanisms.

## 4. Discussions

The COVID-19 pandemic became a global public health issue. Understanding the expression levels and localizations of candidate SARS-CoV-2 receptors in host tissues may provide insights into therapeutics that reduce disease spread, viral replication, disease severity or disease pathology. ACE2 has been implicated in SARS-CoV-2 viral infection 42, 45, 46. Additional host molecules including HSPA5 may also function as receptors for SARS-CoV-2 recognition 15, 43. Thus, the same as ACE2, HSPA5 protein would be closely related to this COVID-19 virus entry, and the distributions and expression levels of this receptor might reflect the susceptibility to the virus and viral replication. However, the impacts of HSPA5 on SARS-CoV-2 susceptibility and the characterization of malignant cancer patients in the COVID-19 outbreaks are unknown. Understanding of the HSPA5 expressions in different normal tissues and malignant tumors is important. In the current study, HSPA5 has been found to be highly expressed in almost all the normal tissues and increased in most tumor tissues, indicating that all the organs will be potentially infected, higher susceptible to SARS-CoV-2 in those with tumors. More importantly, *HSPA5* mRNA levels increase 54.4 fold than that of ACE2 in normal lung, and 253 fold in lung cancer, indicating that HSPA5 should play important roles for SARS-Cov-2 entry in cancer patients through the lungs. This was supported partially by a systematic review of malignant cancers in COVID-19 patients that men and lung cancer were more likely to have the risk of COVID-19, when studied the ACE2 expression 31. Surprisingly, the mRNA expressions of *HSPA5* are much higher than those of *ACE2* in both malignant tumors and normal individuals across almost all kinds of cancer types. Thus, these data implied that, comparing the SARS-CoV-2 that invaded tissues with low expressed ACE2, this virus may more likely invade the highly HSPA5 expressed tissues.

Moreover, patients with malignant tumors are usually weaker and may be more severely affected by SARS-Cov-2. Higher expression of HSPA5 significantly decreased patient survival in OS in 7 types of cancers, including ACC, BLCA, GBM, HNSC, KIRP, LIHC, UVM. Furthermore, our systematic review results indicate that 7.15% of 5,068 COVID-19 cases have malignant cancer coincidental situations, and the rate of more severe events of COVID-19 patients with malignant cancers (33.33%) presented a higher trend than that for all COVID-19 patients (16.09%) with a significant difference (33.33% vs. 16.09%, *p*<0.01). Since almost all cancer tissues had highly expressed HSPA5, this indicated that all tumor patients are susceptible to the SARS-CoV-2 infection, implying the clinical significance of the role of HSPA5 expression. Hence, the susceptibility of malignant cancer patients and the differences of intensity degree could be estimated by exploring HSAP5 expression. Of course, we should point out, in these studies, the number of tumor patients and the sources from different countries were not sufficient and further studies are needed to confirm our findings. The relationship between HSPA5 expression levels of different specific tumor types and the disease severity should further be explored.

Changes in HSPA5 expression levels may affect the susceptibility for virus infection and the severity of COVID-19 disease. For example, decreasing HSPA5 expression would be the potentials to prevent COVID-19, especially those with malignant tumors. Indeed, HSPA5 has been recently implied as an anticancer drug target 8, 110, 111. We might consider the treatment potentials such as using HSPA5 inhibitors 112. Recently virtual screening studies revealed that known HSPA5 inhibitors interferes with the infection by SARS-Cov-2^21^. Two of these drugs, Bosutinib and Ponatinib, are inhibitors of SRC and were patented as also being capable of blocking cell surface HSPA5 expression (http://www.freepatentsonline.com/y2019/0076431.html). Natural products may also interfere with SARS-CoV-2 attachment to stressed cells, which is worth of further investigation 23, 113.

## 5. Conclusions

In summary, our analyses showed that HSPA5 is expressed in almost all the normal tissues and elevated expression in tumor tissues. *HSPA5* mRNA levels increase 253-fold than that of *ACE2* in lung cancer, indicating that HSPA5 migh play more important roles for SARS-Cov-2 entry in cancer patients through the lungs. The rate of more severe events for COVID-19 patients with malignant cancers (33.33%) presented a higher trend than that for all COVID-19 patients (16.09%) with a significant difference. Malignant cancer patients are usually weaker and might be more severely affected by COVID-19. Thus, this virus seems more likely to invade tissues with highly expressed HSPA5. Decreasing HSPA5 expression will provide a strategy potentially to prevent COVID-19, especially those with malignant tumors. Collectively, this study may not only imply the clinical significance of the role of HSPA5 in COVID-19 disease and cancers, but also provide a potential clue for further medical treatments and managements of COVID-19 patients.

HSPA5: heat shock protein family A (Hsp70) member 5; BiP: binding immunoglobulin protein; GRP78: glucose regulating protein 78; SARS-CoV-2: severe acute respiratory syndrome coronavirus 2; COVID-19: coronavirus disease 2019; SARS: severe acute respiratory syndrome; MERS: middle east respiratory syndrome; ARDS: acute respiratory distress syndrome; ACE2: angiotensin-converting enzyme 2; IHC: immunohistochemistry; HPA: Human Protein Atlas; GEPIA: The Gene Expression Profiling Interactive Analysis; GTEx: Genotype Tissue Expression; OS: overall survival.

## Supplementary Material

Supplementary figures.

## Figures and Tables

**Table 1:** *HSPA5* RNA expression in different datasets

Dataset	Value	Unit
HPA	247.6	pTPM
GTEx	275.1	pTPM
FANTOM5	449.0	Scaled Tags Per Million
Consensus	43.6	NX

**Note:** pTPM: protein-transcripts per million; NX: normalized expression.

**Table 2:** *HSPA5* RNA expression in different cells from lung tissue

Cell types	Percentages (%)
Pneumocytes	31.67
Bronchial epithelium	5.00
Endothelial cells	28.33
Macrophages	10.00
Other cell types	25.00

**Note:** Data was normalized to nine samples by HPA RNA-sequencing.

**Table 3:** Summary of number and severity of malignant cancer patients with COVID-19

Total cases	Malignant cancers (%)	Severe events of cancer patients		Total severe events (%)	Resources	References
		Yes (%)	No (%)			
138	10 (7.25)	4 (40)	6 (60)	36 (26.09)	China	PMID: 32031570^29^
41	1 (2.44)	0	1	13 (31.71)	China	PMID: 31986264^27^
1590	18 (1.13)	9 (50)	9 (50)	131 (8.24)	China	PMID: 32066541^47^
641	105 (16.38)	20 (19.05)	85 (80.95)	43 (6.71)	China	PMID: 32345594^48^
1276	28 (2.19)	15 (53.57)	13 (46.43)	56 (4.39)	China	PMID: 32224151^49^
1090	218 (20)	61 (27.98)	157 (72.02)	149 (13.67)	USA	PMID: 32357994^50^
751	232 (30.89)	148 (63.79)	84 (36.21)	314 (41.81)	China	PMID: 32479790^51^
5688	334 (5.87)	37 (11.08)	297 (88.92)	555 (9.76)	USA	PMID: 32330541^52^
10486	892 (8.51)	327 (36.66)	565 (63.34)	3094 (29.51)	Belgium	PMID: 32978251^53^
1878	45 (2.40)	29 (64.44)	16 (35.56)	192 (10.22)	Spain	PMID: 32449128^54^
56	25 (44.64)	9 (36)	16 (64)	14 (25)	Italy	PMID: 32403946^55^
1069	36 (3.37)	15 (41.67)	21 (58.33)	132 (12.35)	España	PMID: 32507536^56^
334	167 (50)	56 (33.53)	111 (66.47)	94 (28.14)	Spain	PMID: 33077708^57^
188	53 (28.19)	32 (60.38)	21 (39.62 )	81 (43.09 )	USA	PMID: 33043705^58^
352	15 (4.26)	4 (26.67)	11 (73.33)	24 (6.82)	Korea	PMID: 32924343^59^
459	52 (11.33)	14 (26.92)	38 (73.08)	63 (13.73)	Iran	PMID: 32908083^60^
1476	29 (1.96)	20 (68.97)	9 (31.03)	757 (51.29)	China	PMID: 32857662^61^
135	4 (2.96)	3 (75)	1 (25)	40 (29.63)	China	PMID: 32198776^62^
336	14 (4.17)	6 (42.86)	8 (57.14)	133 (39.58)	China	PMID: 32883943^63^
2665	109 (4.09)	32 (29.36)	77 (70.64)	293 (10.99)	China	PMID: 32522278^64^
15	10 (66.67)	7 (70)	3 (30)	7 (46.67)	Poland	PMID: 32769026^65^
240	17 (7.08)	11 (64.71)	6 (35.29)	120 (50)	China	PMID: 33120785^66^
64	16 (25)	1 (6.25)	15 (93.75)	12 (18.75)	Turkey	PMID: 32854573^67^
210	5 (2.38)	5 (100)	0	87 (41.43 )	China	PMID: 32641174^68^
276	3 (1.09 )	1 (33.33)	2 (66.67)	14 (5.07)	China	PMID: 32727456^69^
410	22 (5.37)	11 (50)	11 (50)	95 (23.17)	Italy	PMID: 32535188^70^
167	39 (23.35)	16 (41.03)	23 (58.97)	52 (31.14)	Germany	PMID: 32931637^71^
901	22 (2.44 )	1 (4.55)	21 (95.45)	124 (13.76)	China	PMID: 33121497^72^
64	1 (1.56)	1 (100)	0	21 (32.81)	China	PMID: 32741931^73^
1603	122 (7.61)	95 (77.87)	27 (22.13)	192 (11.98)	Italy	PMID: 32579597^74^
204	95 (46.57)	38 (40)	57 (60)	64 (31.37)	Italy	PMID: 32910456^75^
171	116 (67.84)	62 (53.45)	54 (46.55)	73 (42.69)	China	PMID: 33194660^76^
1590	18 (1.13)	7 (38.89)	11 (61.11)	131 (8.24 )	China	PMID: 32396163^77^
217	112 (51.61)	40 (35.71)	72 (64.29)	56 (25.81)	China	PMID: 33192519^78^
2964	17 (0.57)	1 (5.88)	16 (94.12)	239 (8.06)	Iran	PMID: 32353762^79^
1385	98 (7.04)	48 (48.98)	50 (51.02)	293 (21.16)	Spain	PMID: 33172949^80^
26	3 (11.54)	3 (100)	0	19 (73.08)	France	PMID: 32941618^81^
238	2 (0.84)	2 (100)	0	48 (20.17)	China	PMID: 32554861^82^
154	5 (3.25)	2 (40)	3 (60)	2 (1.3)	Turkey	PMID: 33104780^83^
55	52 (94.55 )	24 (46.15)	28 (53.85)	25 (45.45)	UK	PMID: 32678948^84^
716	12 (1.68)	4 (33.33)	8 (66.67)	58 (8.1)	China	PMID: 33033507^85^
67	1 (1.49)	0	1 (100)	13 (19.40)	USA	PMID: 32407719^86^
69	4 (5.80)	1 (25)	3 (75)	14 (20.29)	China	PMID: 32176772^87^
170	23 (13.53)	11 (47.83)	12 (52.17)	90 (52.94)	USA	PMID: 32946859^88^
599	17 (2.84)	1 (5.88)	16 (94.12)	83 (13.86)	China	PMID: 32716901^89^
68	3 (4.41)	3 (100)	0	48 (70.59)	USA	PMID: 32619411^90^
676	33 (4.88)	16 (48.48)	17 (51.52)	140 (20.71)	China	PMID: 33166991^91^
61	3 (4.92)	1 (33.33)	2 (66.67)	22 (36.07)	Iran	PMID:32910458^92^
1226	138 (11.26)	47 (34.06 )	91 (65.94)	330 (26.92)	Italy	PMID: 32707770^93^
299	16 (5.35)	4 (25 )	12 (75)	71 (23.75 )	Intl mctr	PMID: 32616597^94^
5154	312 (6.05)	66 (21.15)	246 (78.85)	484 (9.39)	USA	PMID: 33112411^95^
323	5 (1.55)	1 (20)	4 (80)	26 (8.05)	China	PMID: 32361738^96^
2476	195 (7.88)	63 (32.31)	132 (67.69)	320 (12.92)	USA	PMID: 33142266^97^
120	30 (25)	11 (36.67 )	19 (63.33)	47 (39.17)	UK	PMID: 32968429^98^
105	16 (15.24)	11 (68.75)	5 (31.25)	51 (48.57)	USA	PMID: 32444880^99^
57	24 (42.11)	5 (20.83)	19 (79.17)	7 (12.28)	Switzerland	PMID: 32571972^100^
1480	740 (50)	140 (18.92)	600 (81.08)	225 (15.2)	Turkey	PMID: 32776581^101^
585	117 (20)	29 (24.79)	88 (75.21)	129 (22.05)	USA	PMID: 32986528^102^
3014	100 (3.32)	30 (30)	70 (70)	755 (25.05)	USA	PMID: 32997958^103^
120	7 (5.83 )	5 (71.43)	2 (28.57)	30 (25)	China	PMID: 32279115^104^
483	5 (1.04)	0	5 (100)	62 (12.84)	China	PMID: 32683596^105^
961	21 (2.19)	10 (47.62)	11 (52.38)	242 (25.18)	China	PMID: 32716553^106^
49	6 (12.24)	4 (66.67)	2 (33.33)	38 (77.55)	Israel	PMID: 32923991^107^
10237	76 (0.74 )	9 (11.84)	67 (88.16)	228 (2.23)	Korea	PMID: 33127965^108^
156	2 (1.28)	0	2 (100)	3 (1.92 )	Spain	PMID: 33220760^109^
70874	5068 (7.15)^a^	1689 (33.33)^b^	3379 (66.67)^c^	11404 (16.09)^d^	Total	

**Note:** a: 5068/70874; b: 1689/5068; c: 3379/5068; d: 11404/70874; Severe events include ICU and died cases; Intl mctr: International multicentre.
