# Supplementary material for: Evaluation and characterization of HSPA5 (GRP78) expression profiles in normal individuals and cancer patients with COVID-19
Source: Int J Biol Sci. 2021 Feb 18;17(3):897–910. doi: 10.7150/ijbs.54055 (PMC7975696; doi:10.7150/ijbs.54055)

Supplementary figures

Supplementary figure 1. Homologs of the HSPA5 proteins. A. Conservation for HSPA5 in 20 different species. B. The indicated the conserved domains.

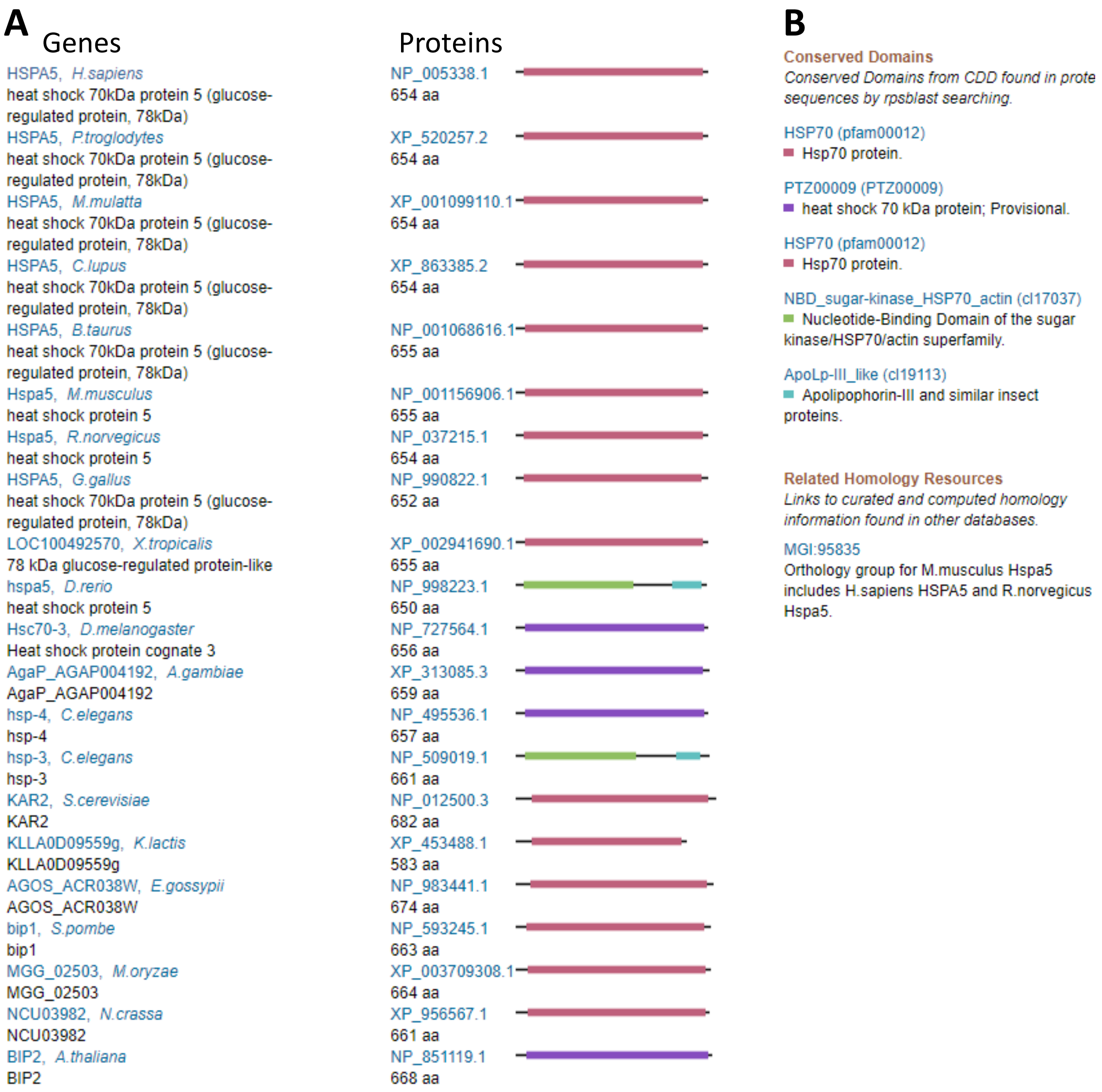

Supplementary figure 2. The *HSPA5* mRNA expression and comparison to *ACE2*. The *HSPA5* mRNA expression information from databases of LUNG - GTEx RNA-seq (A), and LUNG - FANTOM5 CAGE (B) in human lungs. pTPM values give a quantification of the gene abundance which is comparable between different genes and samples. C. *HSPA5* and *ACE2* mRNA levels (NX) in lungs. Consensus dataset of mRNA levels for *HSPA5* and *ACE2* are derived from HPA dataset, GTEx dataset, and FANTOM5 dataset. The RNA-sequencing results generated in the HPA are reported as normalized NX values. In the HPA, a NX value of 1.0 is defined as a threshhold for expression of the corresponding protein. pTPM, transcripts per million. NX, normalized expression.

A

LUNG - GTEx RNA-seq

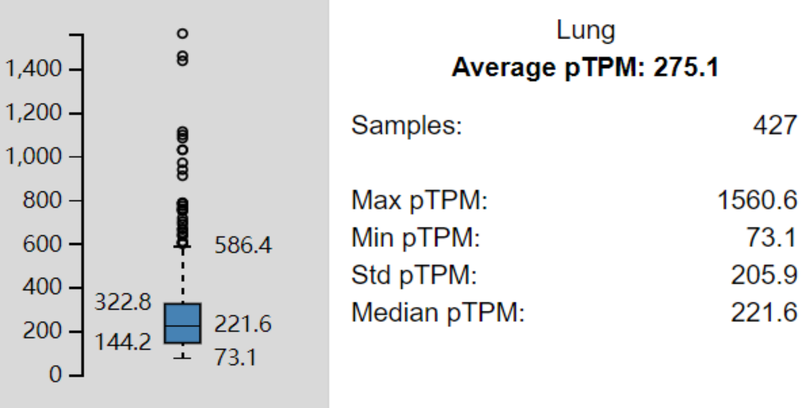

C

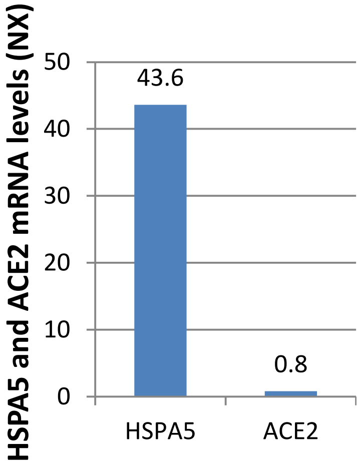

B

LUNG - FANTOM5 CAGE[i]

Max Scaled Tags Per Million	449.0		
	**FANTOM5 sample id**	**Sample description**	**Scaled Tags Per Million**
Lung<br>**Scaled Tags Per Million: 578.7**	FF:10019-101D1	46,65,94 years, mixed	578.7
Lung - right lower lobe<br>**Scaled Tags Per Million: 319.3**	FF:10075-102A3	29 years, male	319.3

Supplementary figure 3. Localization of HSPA5 protein in malignant tumors and cancer cell lines. A~E. The representative IHC images of tumors from cancers of colorectal, breast, prostate, lung, and liver, respectively. The images by antibody staining in 20 different tumors is summarized but not showed all. The protein expressions for HSPA5 from malignant tumors were obtained in the database of the Human Protein Atlas (HPA) (https://www.proteinatlas.org/ENSG00000044574-HSPA5/pathology) F~I. The representative IF staining of epidermoid carcinoma cell line A-431, derived from an 85-year-old female, with HSPA5 antibody (cat #: HPA038845, Sigma-Aldrich) for HSPA5 staining (F), nucleus staining (G), microtubules staining (H), and all merged together (I). The images of human cells give overviews about the subcellular location of HSPA5 protein from immunofluorescence microscopy (https://www.proteinatlas.org/ENSG00000044574-HSPA5/cell#human). The representative immunofluorescent images are displayed. Three different organelle probes with different channels are displayed in the multiple color images - nucleus stained in blue, microtubules in red, and an antibody staining targeting the protein of HSPA5 in green.

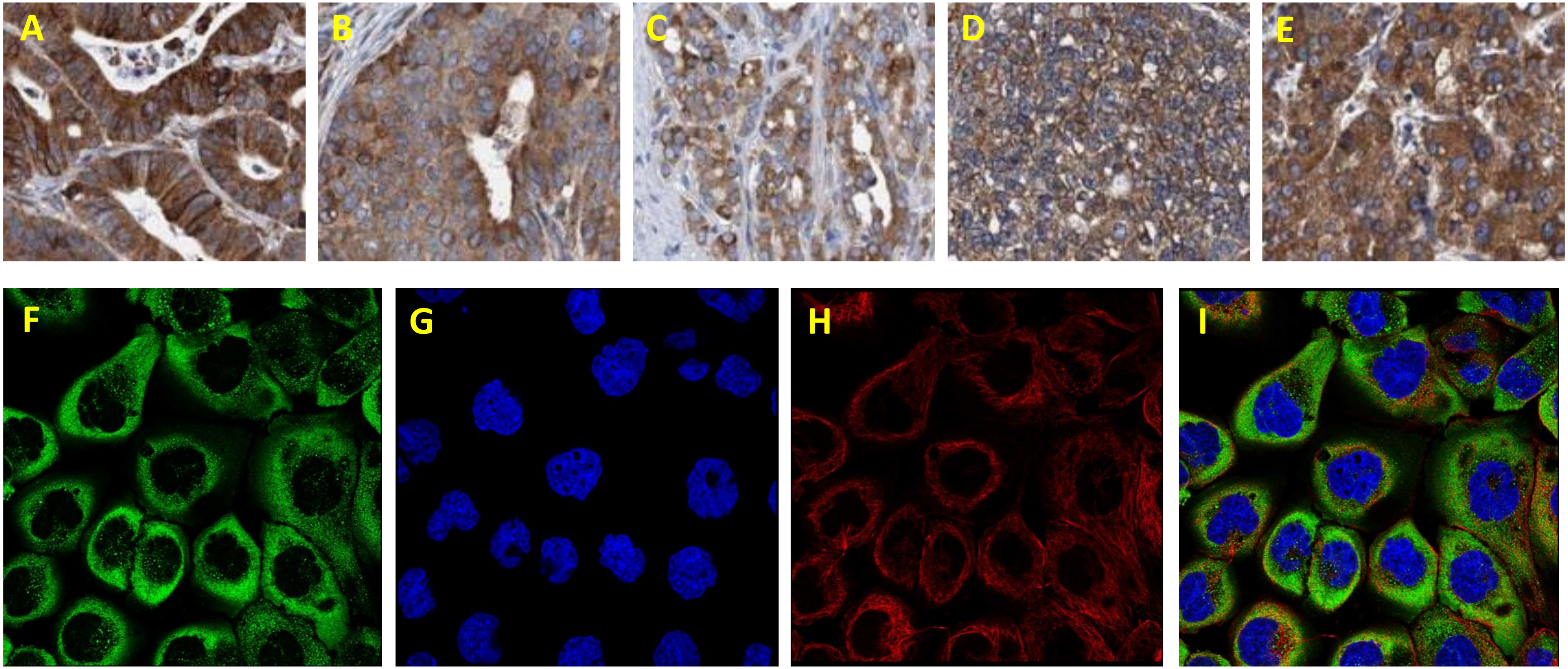

Supplement: Supplementary file 1 — Supplementary figures. [file ijbsv17p0897s1.pdf]
